# Tracking Cancer: Exploring Heart Rate Variability Patterns by Cancer Location and Progression

**DOI:** 10.3390/cancers16050962

**Published:** 2024-02-27

**Authors:** Kfir Ben-David, Harrison L. Wittels, Michael J. Wishon, Stephen J. Lee, Samantha M. McDonald, S. Howard Wittels

**Affiliations:** 1 Department of Surgery, Division of Oncology, Mount Sinai Medical Center, Miami Beach, FL 33140, USA; kfir.bendavid@msmc.com; 2 Department of Surgery, Wertheim School of Medicine, Florida International University, Miami, FL 33199, USA; 3 Tiger Tech Solutions, Inc., Miami, FL 33156, USA; hl@tigertech.solutions (H.L.W.); joe@tigertech.solutions (M.J.W.); smmcdo4@ilstu.edu (S.M.M.); 4 Science, Technology and Research, Inc., Miami, FL 33156, USA; 5 United States Army Research Laboratory, United States Army Combat Capabilities Development Command, Adelphi, MD 20783, USA; stephen.j.lee28.civ@mail.mil; 6 School of Kinesiology and Recreation, Illinois State University, Normal, IL 61761, USA; 7 Department of Anesthesiology, Mount Sinai Medical Center, Miami, FL 33140, USA; 8 Department of Anesthesiology, Wertheim School of Medicine, Florida International University, Miami, FL 33199, USA; 9 Miami Beach Anesthesiology Associates, Miami, FL 33140, USA

**Keywords:** cancer, oncology, heart rate variability, chronic disease, autonomic nervous system, screening

## Abstract

**Simple Summary:**

Heart rate variability, an indicator of autonomic nervous system function, is lower in patients with cancer and worsens with disease progression. What remains less clear is whether the reduced heart rate variability observed in cancer patients is consistent across different cancer locations and their progression. Heart rate variability was measured in 798 patients at a tertiary medical center. This study evaluated patterns in heart rate variability for the most prevalent cancers including breast, gastrointestinal tract, genitourinary, and respiratory system cancers. Our study found that, compared to non-cancer patients, cancer patients exhibited lower heart rate variability that precipitously declined with disease progression across all stages of cancer. Additionally, it appears that this pattern uniformly occurs across all cancers we evaluated. More importantly, the reduced heart rate variability occurred independent of common co-morbidities like cardiovascular diseases, diabetes, and obesity. Our study findings highlight the importance of physicians integrating heart rate variability into routine health assessments and its use as the first step in screening for cancers.

**Abstract:**

Reduced heart rate variability (HRV) is an autonomic nervous system (ANS) response that may indicate dysfunction in the human body. Consistent evidence shows cancer patients elicit lower HRV; however, only select cancer locations were previously evaluated. Thus, the aim of the current study was to explore HRV patterns in patients diagnosed with and in varying stages of the most prevalent cancers. At a single tertiary academic medical center, 798 patients were recruited. HRV was measured via an armband monitor (Warfighter Monitor^TM^, Tiger Tech Solutions, Inc., Miami, FL, USA) equipped with electrocardiographic capabilities and was recorded for 5 to 7 min with patients seated in an upright position. Three time-domain metrics were calculated: SDNN (standard deviation of the NN interval), rMSSD (the root mean square of successive differences of NN intervals), and the percentage of time in which the change in successive NN intervals exceeds 50ms within a measurement (pNN50). Of the 798 patients, 399 were diagnosed with cancer. Cancer diagnoses were obtained via medical records one week following the measurement. Analysis of variance models were performed comparing the HRV patterns between different cancers, cancer stages (I–IV), and demographic strata. A total of 85% of the cancer patients had breast, gastrointestinal, genitourinary, or respiratory cancer. The cancer patients were compared to a control non-cancer patient population with similar patient size and distributions for sex, age, body mass index, and co-morbidities. For all HRV metrics, non-cancer patients exhibited significantly higher rMSSDs (11.1 to 13.9 ms, *p* < 0.0001), SDNNs (22.8 to 27.7 ms, *p* < 0.0001), and pNN50s (6.2 to 8.1%, *p* < 0.0001) compared to stage I or II cancer patients. This significant trend was consistently observed across each cancer location. Similarly, compared to patients with stage III or IV cancer, non-cancer patients possessed lower HRs (−11.8 to −14.0 bpm, *p* < 0.0001) and higher rMSSDs (+31.7 to +32.8 ms, *p* < 0.0001), SDNNs (+45.2 to +45.8 ms), *p* < 0.0001, and pNN50s (19.2 to 21.6%, *p* < 0.0001). The HR and HRV patterns observed did not significantly differ between cancer locations (*p* = 0.96 to 1.00). The depressed HRVs observed uniformly across the most prevalent cancer locations and stages appeared to occur independent of patients’ co-morbidities. This finding highlights the potentially effective use of HRV as a non-invasive tool for determining common cancer locations and their respective stages. More studies are needed to delineate the HRV patterns across different ages, between sexes and race/ethnic groups.

## 1. Introduction

Nearly half of all patients in the US diagnosed with cancer are in the advanced stage, significantly increasing their risks of mortality [1,2,3]. Early detection via screening provides the best opportunity for effective diagnosis, treatment, and consequently improvement in overall cancer survival [4,5,6]. However, most screening procedures often accompany burdensome preparation and/or physical discomfort during testing. These limitations are shown to deter many individuals from adhering to public health screening recommendations for prevalent cancers such as breast and colon cancer [7,8,9,10]. Thus, it is important to explore non-invasive methodologies that may identify cancer in earlier stages, possibly facilitating higher screening adherence, better guidance for early treatment options, and improved survival.

Over the past two decades, studies have consistently demonstrated that heart rate variability (HRV) effectively predicts and tracks certain cancer-related outcomes [11,12,13]. HRV reflects the interplay between the sympathetic and parasympathetic nervous systems (SNS and PNS, respectively [14]), the two branches of the autonomic nervous system (ANS). The ANS regulates many physiological processes, making it an excellent indicator of physiological abnormalities present in the human body. Many studies have previously observed lower HRVs among patients diagnosed with certain cancers like breast, colorectal, and lung [13,15,16,17]. Several studies have posited that the lower HRVs reflect abnormal upregulation of the SNS and/or PNS consequent to the increased innervation of sympathetic and parasympathetic fibers in cancerous tumors. Moreover, these studies have suggested that the upregulation of the SNS and/or PNS may also promote tumor growth and metastasis [18,19]. More importantly, studies have observed larger decrements in HRV among cancer patients in the advanced stages which were associated with poorer prognosis, increased mortality risk, and decreased survivorship following surgical operations and pharmaceutical interventions [13,20,21]. While scientific evidence reliably shows depressed HRV patterns among patients diagnosed with cancer, much less is known about whether specific cancer locations such as gastrointestinal and breast cancer can elicit varying HRV patterns at different stages. In support, individual cancers may affect different parts of the nervous system (e.g., central vs peripheral), different bodily tissues, elicit unique biomarkers, induce varying levels of inflammation, etc., potentially diversifying their influence on the ANS. Currently, a significant drawback of research is the limited number of cancer locations evaluated in a single study. While the few systematic reviews available offer a comprehensive assessment of HRV patterns among multiple cancers, the findings are inconclusive regarding whether HRV differs between cancer locations [13]. This ambiguity is likely related to the higher heterogeneity in study designs, methodologies, and the comparison groups (e.g., healthy, non-cancer, chronic conditions).

Knowing whether cancer-specific HRV patterns exist is critical to the medical community. Using non-invasive, accurate metrics like HRV, treating physicians may be able to identify cancers earlier or even more accurately diagnose and predict prognoses of various cancers and cancers at different stages. To our knowledge, no previous studies have explored patterns of HRV in five different cancer locations at various stages. Therefore, the purpose of this study was to evaluate HRV patterns among a large sample of patients with varying cancer diagnoses. Our objective was to explore potential differential patterns in HRV across cancer locations and stages.

## 2. Materials and Methods

### 2.1. Study Design

This was a cross-sectional study among a diverse patient population with or without a cancer diagnosis. HRV metrics were measured in every patient for up to seven minutes using an armband monitor and occurred prior to the patients’ doctor appointment. HRV patterns were evaluated across all types of cancer, by cancer location, and across cancer stages among patients not yet diagnosed with cancer.

### 2.2. Recruitment and Study Sample

Patients with any scheduled surgical or non-surgical procedures (e.g., colonoscopy, Pap smear, biopsy) and not currently diagnosed with cancer were recruited from a tertiary care academic medical center in the southeastern region of the US from April to December 2023. Prior to their procedure, each patient underwent HRV recording using an armband monitor (Warfighter Monitor^TM^, Tiger Tech Solutions, Inc., Miami, FL, USA). The status of the patients’ cancer diagnosis was blindly extracted from medical records by study personnel one week following the patient’s HRV measurement. We recruited 798 patients, of which 50.0% (n = 399) were diagnosed with cancer, 52.8% were male, and who were predominantly Hispanic (54.3%) and classified as overweight (38.6%) or obese (26.1%). The distributions for most of the demographic characteristics were similar, with no statistical significance observed except for race/ethnicity (*p* < 0.05 with more Hispanic patients diagnosed with cancer (59.4% vs 49.1%, *p* < 0.05)). In the cancer cohort, a majority were diagnosed with breast cancer (18.1%), gastrointestinal cancer (15.0%), genitourinary cancer (27.1%), respiratory cancer (25.1%), and other cancers (14.8%). This group included patients diagnosed with less common cancer types such as neurological and hematological malignancies (see Table 1). All study protocols and procedures followed the principles stated in the Declaration of Helsinki. Patients were fully informed of the study details and voluntarily provided consent.

### 2.3. ANS Function via Heart Rate Variability

Heart rate variability was calculated using electrocardiography in each patient and used to represent ANS function. Patients wore an armband monitor (Warfighter Monitor^TM^, [WFM], Tiger Tech Solutions, Miami, FL, USA) equipped with electrocardiographic capabilities that was previously validated in diverse subpopulations [22,23,24,25]. The WFM was fastened on the upper left arm around the widest part of the biceps muscle of the patient via an elastic strap. At the time of measurement, the patients were seated in an upright position, nearly motionless, and breathing at a normal rate for a period between 5 to 7 min. HRV was quantified using three consecutive minutes of the ECG measurement. For the ECG sampling rate, we utilized 100 Hz which provides sufficient bandwidth to detect QRS peaks bandpass filtered between 8 to 15 Hz [26].

### 2.4. Heart Rate Variability Metrics

Three time-domain HRV metrics were calculated using the changes in the inter-beat intervals. RR intervals were the time between R waves on consecutive QRS complexes and NN intervals were noise-free RR intervals. R peaks were detected utilizing a modified Pan–Tompkins algorithm [27]. Noise-free RR intervals were validated using established signal quality indices (SQIs) [28]. From these data, three separate time-domain indices were derived including SDNN (standard deviation of the NN interval), rMSSD (the root mean square of successive differences between NN intervals), and the percentage of time in which the change in successive NN intervals exceeds 50 ms within a given measurement (pNN50). These HRV time-domain indices have been shown to reflect parasympathetic and sympathetic autonomic output [14,29,30].

### 2.5. Statistical Analysis

The analyses performed aimed to evaluate patterns of HRV among different cancer types and their respective clinical staging. First, we performed a distribution-by-distribution analysis to validate that cancer and non-cancer cases were similar with respect to sex, age, race/ethnicity, body mass index, and presence of co-morbidities (cardiovascular, respiratory, endocrine, neurological, etc.) by matching cancer and non-cancer cases by each stratum of the selected characteristics. Chi-square tests for homogeneity and *t*-tests were used for categorical and continuous variables, respectively. Despite a sample size of 798 patients with 399 having a cancer diagnosis, subdividing the cancer cases into uncommon cancer types resulted in, in some cases, small sample sizes. As such, we grouped the cancer types by their location: breast (n = 72), gastrointestinal (n = 60), genitourinary (n = 108), respiratory (n = 100), and other (n = 59). Additionally, the stages of cancer were categorized into four groups: stage I to stage IV. The metrics used to measure patterns in HRV were rMSSD, SDNN, and pNN50. Two separate analyses were performed. For the first analysis, the HRV patterns were evaluated across three groups: normal (non-cancer patients), non-metastatic, and metastatic and compared via an analysis of variance (ANOVA). For the second analysis, HRV patterns were evaluated for all cancer location groups with and between non-metastatic and metastatic strata via an ANOVA. Multiple comparisons for both analyses were performed, and familywise error was accounted for using Tukey’s test. The a priori alpha level was set at α < 0.05. All statistical analyses were performed in MATLAB, version 2021b (MathWorks, Natick, MA, USA).

## 3. Results

### 3.1. HRV Patterns for All Cancers

Table 2 presents the HR and HRV metrics for the study sample for all cancer cases and specific cancers by cancer stage. Patients with Stages I and II elicited HRs of 69.9 ± 5.5 and 82.4 ± 5.9 bpm, rMSSDs of 40.0 ± 5.5 and 26.4 ± 5.5 ms, SDNNs of 43.8 ± 5.8 and 30.1 ± 5.9 ms, and pNN50s of 33.9 ± 6.0 and 18.2 ± 5.8, respectively. Individuals with advanced cancer (stages III and IV) showed worsened cardiac function with higher HRs: 81.3 ± 4.4 and 88.1 ± 3.5 bpm and lower values for rMSSD: 15.9 ± 2.9 and 9.4 ± 2.7 ms, SDNN: 20.5 ± 2.8 and 14.7 ± 2.5 ms, and pNN50: 14.5 ± 4.9 and 10.4 ± 3.6, respectively. The HR and HRV metrics for specific cancers followed the same trend across cancer stages with greater ANS disturbance occurring in the advanced stages. For all cancer locations, significant differences in HR and HRV metrics were observed between stages I and II, I and III, and I and IV. For rMSSD and SDNN, significant between-stage differences were observed for I vs II, II vs III, I vs III, I vs IV, and II vs IV. Interestingly, less between-stage comparisons were significant for the HR and pNN50 measures.

Within each cancer stage group, however, the HR and HRV metrics appeared similar between the cancer locations.

### 3.2. HRV Patterns between Cancer and Non-Cancer Patients

On average, non-cancer patients elicited a HR of 71.2 ± 8.1 bpm, rMSSD of 45.6 ± 7.9 ms, SDNN of 63.0 ± 11.1 ms, and pNN50 of 33.7 ± 6.9. The mean differences in HR and HRV between patients with and without cancer are shown in Table 3. On average, non-cancer patients elicited significantly lower HRs compared to patients diagnosed with gastrointestinal, genitourinary, respiratory, and other cancers. For stage I cancers of any location, no significant differences in HR, RR interval, or pNN50 were observed between non-cancer patients. However, non-cancer patients exhibited significantly higher HRVs compared to patients diagnosed with stage I breast (rMSSD: +6.1 ms, *p* = 0.003 and SDNN: +18.8 ms, *p* < 0.0001), genitourinary (rMSSD: +4.8, *p* = 0.01 and SDNN: +18.8, *p* < 0.0001), and respiratory cancers (rMSSD: +7.2 ms, *p* < 0.0001 and SDNN: +19.9, *p* < 0.0001). Compared to patients with stage II, III, or IV cancer, regardless of location, non-cancer patients elicited significantly lower HRs (−9.2 to −18.9 bpm, *p* < 0.0001) and higher RR intervals (+113.9 to +186.7 ms, *p* < 0.0001), rMSSDs (+16.4 to +36.4 ms, *p* < 0.0001), SDNNs (+35.3 to +48.7 ms, *p* < 0.0001), and pNN50s (+13.8 to +24.1, *p* < 0.0001). Of note, the mean differences in HR and HRV metrics generally increased in magnitude with advancing stages of cancer. Additionally, the larger magnitudes and significant differences were observed across all advanced cancer locations. 

### 3.3. HRV Patterns by Cancer Location and Stages

In the Supplemental Tables S1–S4, the mean differences in HR and HRV are compared between cancer locations separately for stages I through IV. For patients with non-metastatic cancers (stage I and II), no significant differences in HR (Table S4) were observed between cancer locations. Like HR, for HRV metrics there were no significant differences observed between any cancer locations among patients with non-metastatic cancers (Tables S1–S5). For patients with metastatic cancers (stage III or stage IV), similar observations were made with no significant differences occurring for HR, rMSSD, SDNN, or pNN50 between cancer locations. Graphical representations of these relationships are presented in Figure S1.

**Figure 1:**
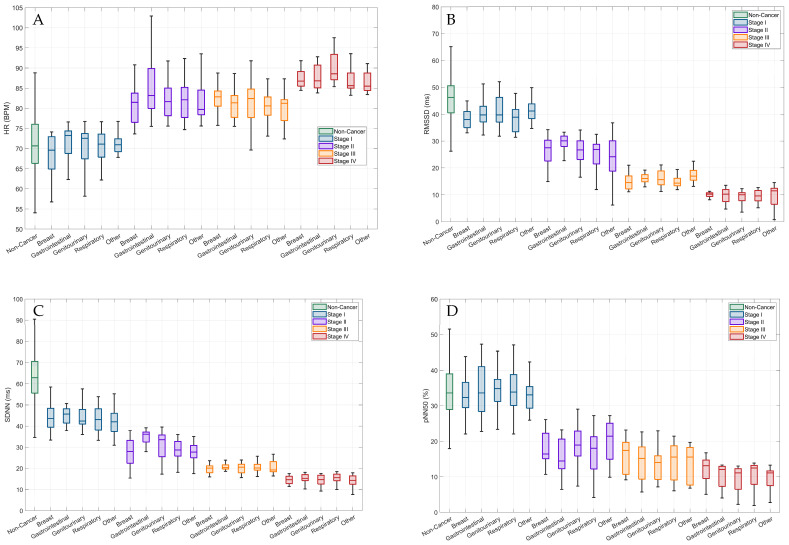
Autonomic nervous system function in cancer and non-cancer cases by cancer location and stage. (**A**) HR (bpm), (**B**) rMSSD (ms), (**C**) SDNN (ms) and (**D**) pNN50 (%).

Figure 1A–D depict trends in HR and HRV for all cancer locations and the major stages of cancer (stage I through IV). For HR, an overall increasing trend was observed for each cancer location across advancing cancer stages, with significant differences observed for stages I, II, III, and IV. For HRV metrics, an overall decreasing trend was observed for each cancer location across stages I, II, III, and IV. Compared to cancer patients, regardless of cancer location, non-cancer patients appeared to exhibit significantly lower HRs and higher HRVs for most stages. 

## 4. Discussion

The purpose of this study was to explore patterns in HRV between various cancer locations at early and late stages in a large sample of cancer versus non-cancer patients. The major findings of this study were that (1) compared to non-cancer patients, patients with cancer exhibited significantly different patterns in HR and HRV, (2) the HR and HRV patterns observed in cancer patients were not location-specific, and (3) the observed depression in HRV and increased HR among cancer patients worsened with advancing clinical stages. 

In the United States and worldwide, cancers of the breast, gastrointestinal tract, genitourinary, and respiratory systems accounted for 3 million deaths in 2020 [2,31]. Our study uniquely observed that patients diagnosed with the most prevalent forms of cancer exhibited significantly reduced HRVs compared to their non-cancer counterparts. The reduced HRVs and increased resting HRs indicate, among cancer patients, altered ANS activity. These patterns have been consistently documented in the scientific literature with studies positing that increased sympathetic drive underlies this abnormal activity of the ANS [13,18,19,20]. Previous studies have suggested that the heightened sympathetic drive promotes tumorigenesis, which was supported by observations of augmented innervation of sympathetic nerve fibers in tumors in addition to higher levels of circulating adrenergic catecholamines [32,33]. Increased inflammation and oxidative stress are also proposed drivers of cancer-related abnormal ANS activity [32]. While other studies observed reduced HRV among cancer patients, the current study possesses several novelties that strengthen and expand upon the existing scientific evidence. First, the HR and HRV patterns were measured in all patients using the same methodology which included real-time electrocardiography. In a recent review by Kloter et al., 2018, which reviewed 19 studies evaluating HRV among various cancer patients, it was found that several types of electrocardiographic-based methodologies were used which employed different monitoring timeframes (10 s to 24 h) and HRV indices, thereby potentially decreasing the confidence in reported results [13]. Additionally, our study assessed HR and HRV patterns prior to a cancer diagnosis in 798 patients. This approach eliminated the potential influence of common pharmaceutical therapies used to treat cancer like chemotherapy, immunotherapy, and radiation, which can alter the inflammatory and oxidative stress associated with cancer. Consequently, these novelties improved the accuracy of evaluating the HR and HRV patterns observed in patients diagnosed with the most prevalent and deadly types of cancer.

The uniformly reduced HRVs observed among cancer patients was significantly different from non-cancer controls matched with similar distributions of prevalent co-morbidities. Many studies evaluating HRV in cancer patients utilized a control group which included healthy individuals [13]. The comparison to a healthy sample is important; however, some of the physiological factors affecting ANS activity in cancer patients like oxidative stress and inflammation are also markers of other serious, life-threatening chronic conditions such as type 2 diabetes mellitus and cardiovascular diseases [33,34,35,36,37]. Therefore, the abnormal ANS activity found among the cancer patients may possibly be, in part, attributed to the presence of another chronic condition(s). Our study, however, consisted of a high prevalence of cardiovascular, respiratory, endocrine, and neurological co-morbidities that were not significantly different between our cancer and non-cancer patients. With this, the lower HRVs and increased HRs observed, uniformly across the cancer locations evaluated, suggests that cancer patients may elicit an altered pattern of ANS activity, potentially unique to those diagnosed with cancer. These results are supported by the few studies previously comparing HRV among cancer patients to individuals with other chronic conditions. For example, Bettermann et al., 2001, among others, found worsened HRV among breast cancer patients compared to individuals with type 2 diabetes [38]. Our study significantly differs as it included multiple cancer locations and evaluated HRV patterns across the major stages, independent of several common co-morbidities.

Interestingly, our study did not observe significant differences in HR nor HRV patterns between cancer locations. In fact, the minimal differences in magnitude in addition to the wide 95% confidence intervals and exceptionally high *p*-values potentially suggests that cancer location does not differentially influence ANS activity but rather affects it in a non-specific manner. This resembles the general adaptation syndrome described by Hans Seyle, where any stressor, regardless of its origin, elicits an increased sympathetic drive and withdrawal of the parasympathetic nervous system [39]. However, in the case of cancer patients, the increased sympathetic drive is chronically sustained. The differences between cancer locations are likely elicited at a more granular physiological level like circulating levels of cancer-specific markers such as prostate-specific antigen and carcinoembryonic antigen, requiring more invasive procedures. However, the consistently lower HRV observed across all cancer locations, independent of select co-morbidities, is encouraging and suggests HRV technology, like the WFM, may serve as an effective initial screening method for cancer.

With advanced stages of cancer, our study observed further reductions in HRV. Patients diagnosed with late-stage cancer (stages III & IV), regardless of cancer location, exhibited significantly higher HRs and lower HRVs. When explored by each cancer stage, an overall linear trend was observed across stages I, II, III, and IV (see Figure 1), with patients diagnosed with stage IV cancer eliciting the highest HRs and lowest values for RR, rMSSD, SDNN, and pNN50. The greater decrements in HRV with advancing cancer appears intuitive given the physiological evidence. In late-stage cancer, tumor metastasis increases the overall cancer burden resulting in exacerbated sympathetic innervation in tumor cells, circulating levels of adrenergic catecholamines, increased inflammation, and oxidative stress [18]. 

The stage-specific pattern for all cancer locations evaluated has been documented in the scientific literature. Kloter et al. found that HRV was significantly lower for patients with metastatic cancer compared to patients with non-metastatic cancer [13]. While previously documented, the current study observed these patterns within a single study and across each major stage of cancer, strengthening the existing evidence. Moreover, our study uniquely evaluated differences between cancer locations within early and late stages; however, no statistically significant differences between cancer locations were observed in either stage. 

### 4.1. Practical Application

Our non-invasive methodology including the electrocardiography and equipment utilized in the current study provides a potential opportunity for increasing adherence to cancer screening recommendations. The WFM armband requires no technicians, extensive set up of preparing for ECG lead placement, nor does it impose a significant time burden on clinicians or patients. While more invasive testing like non-surgical procedures and eventual biopsies are required to confirm a diagnosis for a specific cancer, routinely assessing HR and HRV patterns for the first line of screening has its advantages. For all adults aged ≥45 years, adherence rates for recommended colorectal cancers, with nearly 2 million cases diagnosed per year, ranges between 50 and 70% [40,41]. Routine screenings for colorectal cancer include non-surgical procedures like fecal occult blood testing, sigmoidoscopy, and colonoscopy. Preparation for these tests often induce gastrointestinal discomfort for 24 to 48 h, sedation, reliance on others for transport, and after-procedure care, all of which present significant burdens to screening adherence [7,9,10]. Moreover, a recent report using data derived from the National Cancer Institute (NCI), National Health Information Survey (NHIS), and Behavioral Risk Surveillance Survey (BRFSS) showed that only 14% of breast, colorectal, cervical, and lung cancer diagnoses were detected by the screening procedures recommended for each cancer type [42]. Given this, it is likely that adults may not perceive the physical discomfort and extensive time/resource burden of the screening procedures as “worth it”. Thus, utilizing devices like the WFM as an initial, non-cancer-specific screening procedure may be more effective in helping to identify which patient may be predisposed to cancer and should adhere to the recommended age- and sex-specific cancer screening based on the current study and literature. Additionally, these types of devices are portable, which may reduce the socioeconomic, rural, and race/ethnic inequities in access to these healthcare needs [9,10,31].

### 4.2. Strengths & Limitations

While there are significant strengths to this study, there are a few pitfalls warranting attention. We acknowledge that our analyses did not account for sex, age, race, ethnicity, and body mass index. Analyzing these influential factors for each cancer location and across early and late stages required a substantially larger sample size which is not yet available. The similar distributions in age, sex, BMI, and common co-morbidities observed between the cancer and non-cancer patients significantly increases the confidence in our findings. However, our research group continues collecting ANS data among diverse cancer and non-cancer patients to eventually incorporate and delineate the influences of these characteristics. Another limitation, albeit less critical, is that our study employed a cross-sectional analysis precluding causal inferences; however, it is the most efficient method for addressing the study questions. Future studies should employ a prospective cohort to establish longitudinal patterns more rigorously in HRV and this should be performed across multiple medical centers throughout the United States. 

This is the only study to evaluate, to our knowledge, HR and HRV patterns among patients diagnosed with the most prevalent and deadly cancer cases and recruited from one medical institution. The use of consistent methodologies and protocols among these patients strengthens and expands upon the existing literature. The exclusion of patients with pre-existing cancer or undergoing any pharmaceutical therapies improved the accuracy of our findings by reducing the influences of these factors on ANS activity. Lastly, the WFM used in this study to measure HR and HRV has been previously validated against the gold standard electrocardiographic methodologies [23].

## 5. Conclusions

For the most prevalent cancers, which afflict nearly 20 million patients worldwide, our study identified significantly lower HRV across all major stages. Moreover, these patterns occur potentially independent of common co-morbidities like cardiovascular, respiratory, endocrine, and neurological diseases. While cancer-specific differences in HRV were not observed, the uniform and unique reduction in HRV is encouraging as it highlights the importance of physicians integrating heart rate variability into routine health assessments and its use as the first step in screening for cancers. Additionally, the non-invasive, less technical nature of measuring HRV, especially when using equipment like the WFM, may increase screening adherence via eliminating the physical discomfort and other burdens of current screening methodologies. Further, its portability may improve the inequities in access to healthcare resources. It is acknowledged that measuring HRV is likely one of many parts to diagnosing cancer. However, its information may lead to earlier diagnoses, especially when combined with other metrics leading scientists in developing more precise predictive models using artificial intelligence. The next steps for future research include delineating the patterns in HRV among influential factors like age, sex, race/ethnicity, creating HRV thresholds, and integrating other physiological metrics to produce accurate diagnostic and prognostic cancer screening tools. 

## Figures and Tables

**Table 1:** Distributions of study sample characteristics for cancer and non-cancer cases.

	Cancer Cases (n = 399)	Non-Cancer Cases (n = 399)	Test for Homogeneity **
	Mean (SD) or %	Mean (SD) or %	
Sex (% Male)	50.4	55.1	*p* < 0.00001
Age (years)	68.1 ± 13.2	58.6 ± 15.7	*p* < 0.00001
18–29	1.6	4.0	*p* < 0.00001
30–39	2.5	9.5	*p* < 0.00001
40–49	6.3	11.3	*p* < 0.00001
50–59	8.5	26.1	*p* < 0.00001
60–69	27.1	20.1	*p* < 0.00001
70–79	38.4	22.1	*p* < 0.00001
≥80	15.5	7.0	*p* < 0.00001
Race/Ethnicity			
Non-Hispanic White	33.3	42.1	*p* < 0.00001
Non-Hispanic Black	5.8	7.0	*p* < 0.00001
Hispanic	59.4	49.1	*p* < 0.00001
Other	0.3	0.5	*p* < 0.00001
Body Mass Index (kg/m^2^)	27.5 ± 5.8	27.1 ± 5.3	*p* < 0.0012
BMI Classification (%)			
Normal Weight	29.3	30.3	*p* < 0.00001
Overweight	38.4	38.9	*p* < 0.00001
Obese	27.3	24.8	*p* < 0.00001
Comorbidities (%)			
Cardiovascular	74.7	55.4	*p* < 0.00001
Respiratory	36.3	29.8	*p* < 0.00001
Endocrine	47.6	41.4	*p* < 0.00001
Neurological	33.3	31.3	*p* < 0.00001
Other	57.6	56.6	*p* < 0.00001
Cancer Location (n, %)	399	0	
Breast	18.1	-----	-----
Gastrointestinal	15.0	-----	-----
Genitourinary	27.1	-----	-----
Respiratory	25.1	-----	-----
Other	14.8	-----	-----

SD = standard deviation; BMI = body mass index; ** Chi-square and *t*-tests were performed to assess homogeneity between the cancer and non-cancer cases. The null hypothesis was defined with the assumption that the two samples were heterogeneous. Therefore, a *p* < 0.05 indicates that the samples were homogeneous with respect to the measured sample characteristics. ----- = not applicable given the column references non-cancer patients.

**Table 2:** Comparisons of means (±SD) of heart rate and heart rate variability of cancer patients by cancer location and stage.

	Stage I	Stage II	Stage III	Stage IV
**Heart Rate (bpm)**				
All	69.9 ± 5.5	82.4 ± 5.9	81.3 ± 5.3	88.1 ± 3.5
Breast ^a,d,e^	67.9 ± 6.1	81.3 ± 5.3	82.5 ± 3.8	87.4 ± 2.3
Gastrointestinal ^a,d,e^	71.5 ± 4.2	85.1 ± 8.1	80.9 ± 4.9	87.8 ± 3.2
Genitourinary ^a,c,d,e,f^	69.8 ± 6.5	82.2 ± 4.8	81.9 ± 5.3	90.0 ± 3.9
Respiratory ^a,c,d,e^	70.1 ± 5.2	82.7 ± 6.5	80.7 ± 3.6	87.4 ± 3.6
Other ^a,b,d,e^	70.9 ± 3.6	81.3 ±4.8	80.3 ± 4.1	86.6 ± 2.6
**RR Interval (ms)**				
All	863.5 ± 78.1	731.3 ± 48.6	740.0 ± 40.7	682. ± 26.1
Breast ^a,d,e^	890.7 ± 90.9	740.3 ± 47.8	728.9 ± 33.5	686.8 ± 17.9
Gastrointestinal ^a,d,e^	842.4 ± 52.7	710.8 ± 62.5	744.8 ± 47.4	683.7 ± 24.7
Genitourinary ^a,c,d,e,f^	867.7 ± 95.4	732.4 ± 41.2	735.8 ± 48.2	667.5 ± 28.4
Respiratory ^a,c,d,e^	860.8 ± 74.5	729.8 ± 52.2	744.7 ± 33.5	687.7 ± 27.4
Other ^a,b,d,e^	848.5 ± 44.4	740.6 ± 40.8	748.9 ± 38.1	693.4 ± 20.8
**rMSSD (ms)**				
All	40.0 ± 5.5	26.4 ± 5.5	15.9 ± 2.9	9.4 ± 2.7
Breast ^a,b,d,e,f^	39.4 ± 6.4	26.6 ± 5.2	14.9 ± 2.9	9.6 ± 1.8
Gastrointestinal ^a,b,d,e,f^	40.4 ± 5.2	29.2 ± 3.4	15.9 ± 1.9	9.7 ± 2.7
Genitourinary ^a,b,c,d,e,f^	40.8 ± 5.8	26.7 ± 4.4	16.1 ± 2.9	9.2 ± 2.5
Respiratory ^a,b,c,d,e,f^	38.2 ± 4.9	25.4 ± 5.5	15.4 ± 3.3	9.4 ± 2.2
Other ^a,c,d,e,f^	41.9 ± 5.1	24.4 ± 8.4	17.4 ± 2.7	9.5 ± 4.3
**SDNN (ms)**				
All	43.8 ± 5.8	30.1 ± 5.9	20.5 ± 2.8	14.7 ± 2.5
Breast ^a,d,e,f^	44.2 ± 6.6	27.7 ± 6.3	20.4 ± 3.3	14.7 ± 2.1
Gastrointestinal ^a,b,d,e,f^	44.9 ± 3.9	34.9 ± 3.5	20.6 ± 14.7	15.2 ± 2.2
Genitourinary ^a,b,d,e,f^	44.3 ± 5.2	31.4 ± 6.7	20.2 ± 2.5	14.4 ± 2.4
Respiratory ^a,b,d,e,f^	43.2 ± 6.2	28.8 ± 5.1	20.8 ± 2.9	15.1 ± 2.4
Other ^a,b,d,e,f^	42.3 ± 7.0	27.8 ± 4.7	20.8 ± 5.1	13.9 ± 3.3
**pNN50**				
All	33.9 ± 6.0	18.2 ± 5.8	14.5 ± 4.9	10.4 ± 3.6
Breast ^a,d,e^	32.9 ± 6.1	18.4 ± 5.1	16.1 ± 4.9	12.0 ± 3.8
Gastrointestinal ^a,d,e^	34.3 ± 7.5	15.8 ± 5.5	14.7 ± 5.3	10.2 ± 3.6
Genitourinary ^a,d,e,f^	34.3 ± 5.4	19.0 ± 5.7	13.6 ± 4.6	9.8 ± 3.2
Respiratory ^a,d,e,f^	34.5 ± 6.5	17.6 ± 6.3	14.4 ± 5.2	10.4 ± 3.9
Other ^a,d,e,f^	32.8 ± 4.7	19.9 ± 6.2	13.9 ± 5.1	9.5 ± 3.6

^a^ = statistically significant difference between Stages I and II; ^b^ = statistically significant difference between Stages II and III, ^c^ = statistically significant difference between Stages III and IV; ^d^ = statistically significant difference between Stages I and III; ^e^ = statistically significant difference between Stages I and IV; ^f^ = statistically significant difference between Stages II and IV.

**Table 3:** Mean differences in heart rate and heart rate variability between cancer and non-cancer controls and cancer cases by stage.

	Stage I		Stage II		Stage III		Stage IV	
	Mean Difference (95% CI)	*p*-Value	Mean Difference (95% CI)	*p*-Value	Mean Difference (95% CI)	*p*-Value	Mean Difference (95% CI)	*p*-Value
**Heart Rate (bpm)**								
**Non-Cancer vs.**								
Breast	3.2 (−1.9, 8.4)	0.81	−10.2 (−15.8, −4.7)	<0.0001	−11.3 (−16.3, −6.3)	<0.0001	−16.3 (−22.2, −10.4)	<0.0001
Gastrointestinal	−0.3 (5.9, 5.2)	0.99	−13.9 (−20.2, −7.6)	<0.0001	−9.7 (−15.1, −4.3)	<0.0001	−16.7 (−23.2, −10.2)	<0.0001
Genitourinary	1.3 (−2.9, 5.6)	0.99	−11.0 (−15.8, −6.3)	<0.0001	−10.7 (−14.9, −6.5)	<0.0001	−18.9 (−23.8, −13.9)	<0.0001
Respiratory	1.0 (−3.4, 5.5)	0.99	−11.5 (−16.4, −6.5)	<0.0001	−9.5 (−13.8, −5.3)	<0.0001	−16.2 (−21.4, −11.1)	<0.0001
Other	0.3 (−5.4, 5.9)	0.99	−10.1 (−16.4, −3.8)	<0.0001	−9.2 (−14.7, −3.6)	<0.0001	−15.4 (−21.9, −8.9)	<0.0001
**RR Intervals (ms)**								
**Non-Cancer vs.**								
Breast	−36.4 (−97.1, 24.1)	0.85	113.9 (48.6, 179.2)	<0.0001	125.2 (67.2, 183.2)	<0.0001	167.5 (98.3, 236.6)	<0.0001
Gastrointestinal	11.8 (−53.5, 77.1)	0.99	143.4 (69.5, 217.4)	<0.0001	109.4 (45.8, 173.0)	<0.0001	170.5 (93.7, 247.3)	<0.0001
Genitourinary	−13.5 (−64.1, 37.1)	0.99	121.8 (65.9, 177.6)	<0.0001	118.4 (69.2, 167.6)	<0.0001	186.7 (128.6, 244.7)	<0.0001
Respiratory	−6.5 (−58.7, 45.6)	0.99	124.4 (66.3, 182.4)	<0.0001	109.5 (58.9, 160.1)	<0.0001	166.4 (105.8, 227.1)	<0.0001
Other	5.7 (−61.5, 72.8)	0.99	113.6 (39.6, 187.5)	<0.0001	105.4 (40.1, 170.6)	<0.0001	160.8 (83.9, 237.6)	<0.0001
**rMSSD (ms)**								
**Non-Cancer vs.**								
Breast	6.1 (1.0, 11.3)	0.003	18.9 (13.9, 24.0)	<0.0001	30.7 (25.9, 35.4)	<0.0001	35.9 (30.4, 41.5)	<0.0001
Gastrointestinal	5.2 (−0.6, 10.9)	0.15	16.4 (10.9, 21.8)	<0.0001	29.6 (24.2, 34.8)	<0.0001	35.8 (29.9, 41.8)	<0.0001
Genitourinary	4.8 (0.4, 9.1)	0.01	18.8 (14.6, 23.0)	<0.0001	29.5 (25.5, 33.5)	<0.0001	36.4 (31.7, 41.1)	<0.0001
Respiratory	7.2 (2.7, 11.7)	<0.0001	20.2 (15.9, 24.5)	<0.0001	30.1 (25.9, 34.3)	<0.0001	36.2 (31.4, 40.9)	<0.0001
Other	3.7 (−2.1, 9.4)	0.78	21.2 (15.6, 26.8)	<0.0001	28.1 (22.7, 33.5)	<0.0001	36.1 (30.1, 42.0)	<0.0001
**SDNN (ms)**								
**Non-Cancer vs.**								
Breast	18.8 (12.6, 25.0)	<0.0001	35.3 (28.6, 41.9)	<0.0001	42.6 (36.1, 49.1)	<0.0001	48.3 (41.9, 54.7)	<0.0001
Gastrointestinal	18.1 (11.2, 24.9)	<0.0001	28.2 (20.9, 35.5)	<0.0001	42.5 (35.2, 49.8)	<0.0001	47.8 (40.9, 54.7)	<0.0001
Genitourinary	18.8 (13.6, 23.9)	<0.0001	31.6 (25.9, 37.3)	<0.0001	42.9 (37.5, 48.3)	<0.0001	48.7 (43.3, 54.1)	<0.0001
Respiratory	19.9 (14.4, 25.3)	<0.0001	34.2 (28.4, 40.1)	<0.0001	42.2 (36.5, 47.9)	<0.0001	47.9 (42.4, 53.4)	<0.0001
Other	20.8 (13.7, 27.9)	<0.0001	35.2 (27.9, 42.5)	<0.0001	42.3 (34.9, 49.6)	<0.0001	49.1 (42.1, 56.2)	<0.0001
**pNN50**								
**Non-Cancer vs.**								
Breast	0.7 (−4.2, 5.6)	0.99	15.3 (9.9, 20.6)	<0.0001	17.5 (12.9, 22.3)	<0.0001	21.7 (16.1, 27.3)	<0.0001
Gastrointestinal	−0.6 (−6.0, 4.9)	0.99	17.9 (12.1, 23.7)	<0.0001	19.0 (13.9, 24.2)	<0.0001	23.5 (17.2, 29.7)	<0.0001
Genitourinary	−0.6 (−4.7, 3.5)	0.99	14.6 (10.1, 19.2)	<0.0001	20.1 (16.1, 24.1)	<0.0001	23.9 (19.1, 28.6)	<0.0001
Respiratory	0.9 (−5.2, 3.4)	0.99	16.0 (11.4, 20.7)	<0.0001	19.2 (15.1, 23.4)	<0.0001	23.3 (18.3, 28.2)	<0.0001
Other	0.9 (−4.7, 6.5)	0.99	13.8 (8.0, 19.6)	<0.0001	19.7 (14.4, 25.0)	<0.0001	24.1 (17.9, 30.4)	<0.0001

CI = confidence interval; ms = milliseconds; *p*-values reference the comparisons in HR, RR, rMSSD, SDNN, and pNN50 between each cancer location relative to the non-cancer cases.

## Data Availability

The data can be provided by Tiger Tech Solutions, Inc., pending scientific review and a completed materials transfer agreement. Requests for the data should be submitted to the corresponding author: Samantha M McDonald, smmcdo4@ilstu.edu.

## Supplementary Materials

The following supporting information can be downloaded at: https://www.mdpi.com/article/10.3390/cancers16050962/s1, Figure S1: RR Intervals between Cancer and Non-Cancer Cases by Cancer Location and Stage; Table S1: Mean Differences in *Heart Rate* Within Cancer Stages and Between Cancer Locations; Table S2: Mean Differences in *RR Intervals* Within Cancer Stages and Between Cancer Locations; Table S3: Mean Differences in *rMSSD* Within Cancer Stages and Between Cancer Locations; Table S4: Mean Differences in *SDNN* Within Cancer Stages and Between Cancer Locations; Table S5: Mean Differences in *pNN50* Within Cancer Stages and Between Cancer Locations.
