# Supplementary material for: Tracking Cancer: Exploring Heart Rate Variability Patterns by Cancer Location and Progression
Source: Cancers (Basel). 2024 Feb 27;16(5):962. doi: 10.3390/cancers16050962 (PMC10931286; doi:10.3390/cancers16050962)
Supplement: Supplementary file 1 [file cancers-16-00962-s001.zip › cancers-2859810-supplementary.pdf]

Table S1. Mean Differences in ***Heart Rate*** Within Cancer Stages and Between Cancer Locations.

	**Stage I**		**Stage II**		**Stage III**		**Stage IV**	
	Mean Difference 95% CI	p-value	Mean Difference 95% CI	p-value	Mean Difference 95% CI	p-value	Mean Difference 95% CI	p-value
**Cancer Location**								
Breast								
Gastrointestinal	-3.5 (-10.8, 3.7)	0.98	-3.7 (-11.8, 4.4)	0.99	1.6 (-5.4, 8.6)	0.99	-0.4 (-8.9, 8.1)	0.99
Genitourinary	-1.9 (-8.2, 4.5)	0.99	-0.8 (-7.8, 6.2)	0.99	0.6 (-5.5, 6.7)	0.99	-2.6 (-9.9, 4.8)	0.99
Respiratory	-2.2 (-8.6, 4.3)	0.99	1.3 (-8.4, 5.8)	0.99	1.7 (-4.4, 7.9)	0.99	0.04 (-7.5, 7.6)	0.99
Other	-2.9 (-10.3, 4.4)	0.99	0.1 (-7.9, 8.2)	0.99	2.2 (-4.9, 9.3)	0.99	0.8 (-7.7, 9.3)	0.99
Gastrointestinal								
Breast	-3.5 (-10.8, 3.7)	0.98	-3.7 (-11.8, 4.4)	0.99	1.6 (-5.4, 8.6)	0.99	-0.4 (-8.9, 8.1)	0.99
Genitourinary	1.6 (-5.0, 8.3)	0.99	2.9 (-4.7, 10.5)	0.99	-1.0 (-7.5, 5.5)	0.99	-2.2 (10.0, 5.7)	0.99
Respiratory	1.3 (-5.4, 8.1)	0.99	2.4 (-5.3, 10.1)	0.99	0.1 (-6.4, 6.7)	0.99	0.5 (-7.6, 8.5)	0.99
Other	0.6 (-7.1, 8.2)	0.99	3.8 (-4.8, 12.4)	0.99	0.5 (-6.9, 7.9)	0.99	1.3 (-7.7, 10.2)	0.99
Genitourinary								
Breast	-1.9 (-8.2, 4.5)	0.99	-0.8 (-7.8, 6.2)	0.99	0.6 (-5.5, 6.7)	0.99	-2.6 (-9.9, 4.8)	0.99
Gastrointestinal	1.6 (-5.0, 8.3)	0.99	2.9 (-4.7, 10.5)	0.99	-1.0 (-7.5, 5.5)	0.99	-2.2 (10.0, 5.7)	0.99
Respiratory	-0.3 (-6.0, 5.5)	0.99	-0.5 (-6.9, 6.0)	0.99	1.1 (-4.4, 6.7)	0.99	2.7 (-4.1, 9.5)	0.99
Other	-1.1 (-7.9, 5.8)	0.99	0.9 (-6.7, 8.5)	0.99	1.6 (-5.0, 8.2)	0.99	3.4 (-4.5, 11.3)	0.99
Respiratory								
Breast	-2.2 (-8.6, 4.3)	0.99	1.3 (-8.4, 5.8)	0.99	1.7 (-4.4, 7.9)	0.99	0.04 (-7.5, 7.6)	0.99
Gastrointestinal	1.3 (-5.4, 8.1)	0.99	2.4 (-5.3, 10.1)	0.99	0.1 (-6.4, 6.7)	0.99	0.5 (-7.6, 8.5)	0.99
Genitourinary	-0.3 (-6.0, 5.5)	0.99	-0.5 (-6.9, 6.0)	0.99	1.1 (-4.4, 6.7)	0.99	2.7 (-4.1, 9.5)	0.99
Other	-0.7 (-7.6, 6.1)	0.99	1.4 (-6.2, 9.1)	0.99	0.4 (-6.3, 7.1)	0.99	0.8 (-7.3, 8.8)	0.99
Other								
Breast	-2.9 (-10.3, 4.4)	0.99	0.1 (-7.9, 8.2)	0.99	2.2 (-4.9, 9.3)	0.99	0.8 (-7.7, 9.3)	0.99
Gastrointestinal	0.6 (-7.1, 8.2)	0.99	3.8 (-4.8, 12.4)	0.99	0.5 (-6.9, 7.9)	0.99	1.3 (-7.7, 10.2)	0.99

Genitourinary	-1.1 (-7.9, 5.8)	0.99	0.9 (-6.7, 8.5)	0.99	1.6 (-5.0, 8.2)	0.99	3.4 (-4.5, 11.3)	0.99
Respiratory	-0.7 (-7.6, 6.1)	0.99	1.4 (-6.2, 9.1)	0.99	0.4 (-6.3, 7.1)	0.99	0.8 (-7.3, 8.8)	0.99

CI = confidence interval; ms = milliseconds; p-values reference the comparisons in HR, RR, rMSSD, SDNN and pNN50 between different cancer locations.

Table S2. Mean Differences in ***RR Intervals*** Within Cancer Stages and Between Cancer Locations.

	**Stage I**		**Stage II**		**Stage III**		**Stage IV**	
	Mean Difference 95% CI	p-value	Mean Difference 95% CI	p-value	Mean Difference 95% CI	p-value	Mean Difference 95% CI	p-value
**Cancer Location**								
Breast								
Gastrointestinal	48.2 (-37.0, 133.5)	0.91	29.5 (-65.7, 124.7)	0.99	-15.8 (-97.9, 66.3)	0.99	3.0 (-97.0, 103.1)	0.99
Genitourinary	22.9 (-51.6, 97.5)	0.99	7.9 (-74.0, 89.9)	0.99	-6.9 (-78.4, 64.7)	0.99	19.2 (-67.3, 105.7)	0.99
Respiratory	29.9 (-45.7, 105.7)	0.99	10.5 (-72.9, 93.9)	0.99	-15.7 (-88.2, 56.8)	0.99	-0.99 (-89.3, 87.3)	0.99
Other	42.2 (-44.5, 128.8)	0.98	-0.33 (-95.5, 94.9)	0.99	-19.9 (-103.3, 63.6)	0.99	-6.7 (-106.8, 93.4)	0.99
Gastrointestinal								
Breast	48.2 (-37.0, 133.5)	0.91	29.5 (-65.7, 124.7)	0.99	-15.8 (-97.9, 66.3)	0.99	3.0 (-97.0, 103.1)	0.99
Genitourinary	-25.3 (-103.7, 53.2)	0.99	-21.6 (-110.6, 67.3)	0.99	8.9 (-67.2, 85.1)	0.99	16.2 (-76.6, 108.9)	0.99
Respiratory	-18.3 (-97.8, 61.1)	0.99	-19.1 (-109.5, 71.4)	0.99	0.1 (-76.9, 77.2)	0.99	-4.0 (-98.4, 90.3)	0.99
Other	-6.1 (-96.1, 83.9)	0.99	-29.9 (-131.2, 71.5)	0.99	-4.1 (-91.5, 83.3)	0.99	-9.7 (-115.2, 95.8)	0.99
Genitourinary								
Breast	22.9 (-51.6, 97.5)	0.99	7.9 (-74.0, 89.9)	0.99	-6.9 (-78.4, 64.7)	0.99	19.2 (-67.3, 105.7)	0.99
Gastrointestinal	-25.3 (-103.7, 53.2)	0.99	-21.6 (-110.6, 67.3)	0.99	8.9 (-67.2, 85.1)	0.99	16.2 (-76.6, 108.9)	0.99
Respiratory	6.9 (-60.9, 74.9)	0.99	2.6 (-73.7, 78.9)	0.99	-8.8 (-74.5, 56.8)	0.99	-20.2 (.100.0, 59.6)	0.99
Other	19.2 (-60.8, 99.2)	0.99	-8.2 (-97.2, 80.8)	0.99	-13.0 (-90.6, 64.6)	0.99	-25.9 (-118.6, 66.9)	0.99
Respiratory								
Breast	29.9 (-45.7, 105.7)	0.99	10.5 (-72.9, 93.9)	0.99	-15.7 (-88.2, 56.8)	0.99	-0.99 (-89.3, 87.3)	0.99
Gastrointestinal	-18.3 (-97.8, 61.1)	0.99	-19.1 (-109.5, 71.4)	0.99	0.1 (-76.9, 77.2)	0.99	-4.0 (-98.4, 90.3)	0.99
Genitourinary	6.9 (-60.9, 74.9)	0.99	2.6 (-73.7, 78.9)	0.99	-8.8 (-74.5, 56.8)	0.99	-20.2 (.100.0, 59.6)	0.99
Other	12.2 (-68.7, 93.2)	0.99	-10.8 (-101.2, 79.6)	0.99	-4.2 (-82.6, 74.3)	0.99	-25.9 (-118.6, 66.9)	0.99
Other								
Breast	42.2 (-44.5, 128.8)	0.98	-0.33 (-95.5, 94.9)	0.99	-19.9 (-103.3, 63.6)	0.99	-6.7 (-106.8, 93.4)	0.99
Gastrointestinal	-6.1 (-96.1, 83.9)	0.99	-29.9 (-131.2, 71.5)	0.99	-4.1 (-91.5, 83.3)	0.99	-9.7 (-115.2, 95.8)	0.99

Genitourinary	19.2 (-60.8, 99.2)	0.99	-8.2 (-97.2, 80.8)	0.99	-13.0 (-90.6, 64.6)	0.99	-25.9 (-118.6, 66.9)	0.99
Respiratory	12.2 (-68.7, 93.2)	0.99	-10.8 (-101.2, 79.6)	0.99	-4.2 (-82.6, 74.3)	0.99	-5.7 (-100.0, 88.7)	0.99

CI = confidence interval; ms = milliseconds; p-values reference the comparisons in HR, RR, rMSSD, SDNN and pNN50 between different cancer locations.

Table S3. Mean Differences in ***rMSSD*** Within Cancer Stages and Between Cancer Locations.

	**Stage I**		**Stage II**		**Stage III**		**Stage IV**	
	Mean Difference 95% CI	p-value	Mean Difference 95% CI	p-value	Mean Difference 95% CI	p-value	Mean Difference 95% CI	p-value
**Cancer Location**								
Breast								
Gastrointestinal	-0.9 (-8.4, 6.5)	0.99	-2.6 (-9.7, 4.4)	0.99	-1.1 (7.8, 5.6)	0.99	-0.1 (-8.0, 7.8)	0.99
Genitourinary	-1.4 (-7.8, 5.0)	0.99	-0.2 (-6.3, 6.0)	0.99	-1.2 (-6.9, 4.6)	0.99	0.4 (-6.5, 7.4)	0.99
Respiratory	1.1 (-5.4, 7.6)	0.99	1.2 (-5.1, 7.5)	0.99	-0.5 (-6.4, 5.4)	0.99	0.2 (-6.5, 7.4)	0.99
Other	-2.5 (-9.9, 4.9)	0.99	2.2 (-5.0, 9.4)	0.99	-2.6 (-9.4, 4.3)	0.99	0.1 (-7.8, 8.0)	0.99
Gastrointestinal								
Breast	-0.9 (-8.4, 6.5)	0.99	-2.6 (-9.7, 4.4)	0.99	-1.1 (7.8, 5.6)	0.99	-0.1 (-8.0, 7.8)	0.99
Genitourinary	-0.4 (-7.3, 6.5)	0.99	2.5 (-4.1, 8.9)	0.99	-0.1 (-6.3, 6.2)	0.99	0.5 (-6.7, 7.8)	0.99
Respiratory	2.1 (-4.9, 9.0)	0.99	3.8 (-2.8, 10.5)	0.89	0.6 (-5.8, 6.9)	0.99	0.3 (-7.0, 7.7)	0.99
Other	-1.5 (-9.4, 6.4)	0.99	4.8 (-2.7, 12.3)	0.76	-1.4 (-8.7, 5.8)	0.99	0.2 (-7.9, 8.4)	0.99
Genitourinary								
Breast	-1.4 (-7.8, 5.0)	0.99	-0.2 (-6.3, 6.0)	0.99	-1.2 (-6.9, 4.6)	0.99	0.4 (-6.5, 7.4)	0.99
Gastrointestinal	-0.4 (-7.3, 6.5)	0.99	2.5 (-4.1, 8.9)	0.99	-0.1 (-6.3, 6.2)	0.99	0.5 (-6.7, 7.8)	0.99
Respiratory	2.5 (-3.4, 8.4)	0.99	1.4 (-4.3, 7.1)	0.99	0.7 (-4.7, 6.0)	0.99	-0.2 (-6.6, 6.1)	0.99
Other	-1.1 (-8.0, 5.8)	0.99	2.4 (-4.3, 9.0)	0.99	-1.4 (-7.7, 5.0)	0.99	-0.3 (-7.6, 6.9)	0.99
Respiratory								
Breast	1.1 (-5.4, 7.6)	0.99	1.2 (-5.1, 7.5)	0.99	-0.5 (-6.4, 5.4)	0.99	0.2 (-6.5, 7.4)	0.99
Gastrointestinal	2.1 (-4.9, 9.0)	0.99	3.8 (-2.8, 10.5)	0.89	0.6 (-5.8, 6.9)	0.99	0.3 (-7.0, 7.7)	0.99
Genitourinary	2.5 (-3.4, 8.4)	0.99	1.4 (-4.3, 7.1)	0.99	0.7 (-4.7, 6.0)	0.99	-0.2 (-6.6, 6.1)	0.99
Other	-3.6 (-10.6, 3.4)	0.97	0.9 (-5.8, 7.7)	0.99	-2.0 (-8.5, 4.5)	0.99	-0.1 (-7.4, 7.3)	0.99
Other								
Breast	-2.5 (-9.9, 4.9)	0.99	2.2 (-5.0, 9.4)	0.99	-2.6 (-9.4, 4.3)	0.99	0.1 (-7.8, 8.0)	0.99
Gastrointestinal	-1.5 (-9.4, 6.4)	0.99	4.8 (-2.7, 12.3)	0.76	-1.4 (-8.7, 5.8)	0.99	0.2 (-7.9, 8.4)	0.99

Genitourinary	-1.1 (-8.0, 5.8)	0.99	2.4 (-4.3, 9.0)	0.99	-1.4 (-7.7, 5.0)	0.99	-0.3 (-7.6, 6.9)	0.99
Respiratory	-3.6 (-10.6, 3.4)	0.97	0.9 (-5.8, 7.7)	0.99	-2.0 (-8.5, 4.5)	0.99	-0.1 (-7.4, 7.3)	0.99

CI = confidence interval; ms = milliseconds; p-values reference the comparisons in HR, RR, rMSSD, SDNN and pNN50 between different cancer locations

Table S4. Mean Differences in *SDNN* Within Cancer Stages and Between Cancer Locations.

	**Stage I**		**Stage II**		**Stage III**		**Stage IV**	
	Mean Difference 95% CI	p-value	Mean Difference 95% CI	p-value	Mean Difference 95% CI	p-value	Mean Difference 95% CI	p-value
**Cancer Location**								
Breast								
Gastrointestinal	-0.7 (-9.6, 8.2)	0.99	-7.1 (-16.6, 2.4)	0.47	-0.2 (-9.5, 9.3)	0.99	-0.5 (-9.5, 8.5)	0.99
Genitourinary	-0.03 (-7.7, 7.6)	0.99	-3.6 (-12.1, 4.7)	0.99	0.3 (-7.8, 8.3)	0.99	0.3 (-7.6, 8.3)	0.99
Respiratory	1.1 (-6.8, 8.9)	0.99	-1.1 (-9.5, 7.4)	0.99	-0.4 (-8.7, 7.8)	0.99	-0.4 (-8.4, 7.6)	0.99
Other	1.9 (-7.1, 11.0)	0.99	-0.1 (-9.7, 9.4)	0.99	-0.4 (-9.8, 9.1)	0.99	0.8 (-8.3, 9.9)	0.99
Gastrointestinal								
Breast	-0.7 (-9.6, 8.2)	0.99	-7.1 (-16.6, 2.4)	0.47	-0.2 (-9.5, 9.3)	0.99	-0.5 (-9.5, 8.5)	0.99
Genitourinary	0.7 (-7.5, 8.8)	0.99	3.4 (-5.4, 12.3)	0.99	0.4 (-8.3, 9.1)	0.99	0.8 (-7.5, 9.2)	0.99
Respiratory	1.8 (-6.6, 10.1)	0.99	6.1 (-2.9, 15.0)	0.67	-0.3 (-9.1, 8.6)	0.99	0.1 (-8.3, 8.5)	0.99
Other	2.7 (-6.8, 12.2)	0.99	7.0 (-2.9, 17.0)	0.60	-0.2 (-10.2, 9.8)	0.99	1.3 (-8.2, 10.8)	0.99
Genitourinary								
Breast	-1.9 (-8.2, 4.5)	0.99	-3.6 (-12.1, 4.7)	0.99	0.3 (-7.8, 8.3)	0.99	0.3 (-7.6, 8.3)	0.99
Gastrointestinal	0.7 (-7.5, 8.8)	0.99	3.4 (-5.4, 12.3)	0.99	0.4 (-8.3, 9.1)	0.99	0.8 (-7.5, 9.2)	0.99
Respiratory	1.1 (-5.9, 8.1)	0.99	2.6 (-5.1, 10.3)	0.99	-0.6 (-8.1, 8.1)	0.99	-0.8 (-8.0, 6.5)	0.99
Other	2.0 (-6.4, 10.3)	0.99	3.6 (-5.3, 12.5)	0.99	-0.6 (-9.3, 8.1)	0.99	0.5 (-8.1, 8.9)	0.99
Respiratory								
Breast	-2.2 (-8.6, 4.3)	0.99	-1.1 (-9.5, 7.4)	0.99	-0.4 (-8.7, 7.8)	0.99	-0.4 (-8.4, 7.6)	0.99
Gastrointestinal	1.8 (-6.6, 10.1)	0.99	6.1 (-2.9, 15.0)	0.67	-0.3 (-9.1, 8.6)	0.99	0.1 (-8.3, 8.5)	0.99
Genitourinary	1.1 (-5.9, 8.1)	0.99	2.6 (-5.1, 10.3)	0.99	-0.6 (-8.1, 8.1)	0.99	-0.8 (-8.0, 6.5)	0.99
Other	0.9 (-7.6, 9.4)	0.99	0.9 (-8.0, 9.9)	0.99	0.04 (-8.9, 8.9)	0.99	1.22 (-7.4, 9.8)	0.99
Other								
Breast	-2.9 (-10.3, 4.4)	0.99	-0.1 (-9.7, 9.4)	0.99	-0.4 (-9.8, 9.1)	0.99	0.8 (-8.3, 9.9)	0.99
Gastrointestinal	2.7 (-6.8, 12.2)	0.99	7.0 (-2.9, 17.0)	0.60	-0.2 (-10.2, 9.8)	0.99	1.3 (-8.2, 10.8)	0.99
Genitourinary	2.0 (-6.4, 10.3)	0.99	3.6 (-5.3, 12.5)	0.99	-0.6 (-9.3, 8.1)	0.99	0.5 (-8.1, 8.9)	0.99

Respiratory	0.9 (-7.6, 9.4)	0.99	0.9 (-8.0, 9.9)	0.99	0.04 (-8.9, 8.9)	0.99	1.22 (-7.4, 9.8)	0.99

CI = confidence interval; ms = milliseconds; p-values reference the comparisons in HR, RR, rMSSD, SDNN and pNN50 between different cancer locations.

Table S5. Mean Differences in ***pNN50*** Within Cancer Stages and Between Cancer Locations.

	**Stage I**		**Stage II**		**Stage III**		**Stage IV**	
	Mean Difference 95% CI	p-value	Mean Difference 95% CI	p-value	Mean Difference 95% CI	p-value	Mean Difference 95% CI	p-value
**Cancer Location**								
Breast								
Gastrointestinal	-1.3 (-8.3, 5.8)	0.99	2.7 (-4.9, 10.2)	0.99	1.5 (-5.2, 8.1)	0.99	1.8 (-6.3, 9.9)	0.99
Genitourinary	-1.3 (-7.3, 4.8)	0.99	-0.6 (-7.3, 6.0)	0.99	2.5 (-3.3, 8.3)	0.99	2.2 (-4.8, 9.2)	0.99
Respiratory	-1.5 (-7.7, 4.6)	0.99	0.8 (-5.9, 7.5)	0.99	1.7 (-4.2, 7.6)	0.99	1.6 (-5.5, 8.8)	0.99
Other	0.2 (-6.9, 7.3)	0.99	-1.4 (-9.0, 6.1)	0.99	2.1 (-4.6, 8.9)	0.99	2.5 (-5.6, 10.6)	0.99
Gastrointestinal								
Breast	-1.3 (-8.3, 5.8)	0.99	2.7 (-4.9, 10.2)	0.99	1.5 (-5.2, 8.1)	0.99	1.8 (-6.3, 9.9)	0.99
Genitourinary	0.02 (-6.5, 6.5)	0.99	-3.3 (-10.3, 3.8)	0.99	1.0 (-5.1, 7.2)	0.99	0.4 (-7.8, 7.5)	0.99
Respiratory	-0.3 (-6.9, 6.4)	0.99	-1.9 (-9.0, 5.2)	0.99	0.2 (-6.0, 6.5)	0.99	-0.2 (-7.8, 7.5)	0.99
Other	1.5 (-6.1, 9.0)	0.99	-4.1 (-12.0, 3.8)	0.99	0.7 (-6.4, 7.8)	0.99	0.7 (-7.9, 9.3)	0.99
Genitourinary								
Breast	-1.3 (-7.3, 4.8)	0.99	-0.6 (-7.3, 6.0)	0.99	2.5 (-3.3, 8.3)	0.99	2.2 (-4.8, 9.2)	0.99
Gastrointestinal	0.02 (-6.5, 6.5)	0.99	-3.3 (-10.3, 3.8)	0.99	1.0 (-5.1, 7.2)	0.99	0.4 (-7.8, 7.5)	0.99
Respiratory	-0.3 (-5.8, 5.3)	0.99	1.4 (-4.7, 7.5)	0.99	-0.8 (-6.1, 4.5)	0.99	-0.6 (-7.0, 5.9)	0.99
Other	1.5 (-5.2, 8.1)	0.99	-0.8 (-7.9, 6.2)	0.99	-0.4 (-6.7, 5.9)	0.99	0.3 (-7.2, 7.8)	0.99
Respiratory								
Breast	-1.5 (-7.7, 4.6)	0.99	0.8 (-5.9, 7.5)	0.99	1.7 (-4.2, 7.6)	0.99	1.6 (-5.5, 8.8)	0.99
Gastrointestinal	-0.3 (-6.9, 6.4)	0.99	-1.9 (-9.0, 5.2)	0.99	0.2 (-6.0, 6.5)	0.99	-0.2 (-7.8, 7.5)	0.99
Genitourinary	-0.3 (-5.8, 5.3)	0.99	1.4 (-4.7, 7.5)	0.99	-0.8 (-6.1, 4.5)	0.99	-0.6 (-7.0, 5.9)	0.99
Other	1.7 (-5.0, 8.5)	0.99	-2.2 (-9.3, 4.9)	0.99	0.5 (-5.9, 6.8)	0.99	0.9 (-6.8, 8.5)	0.99
Other								
Breast	0.2 (-6.9, 7.3)	0.99	-1.4 (-9.0, 6.1)	0.99	2.1 (-4.6, 8.9)	0.99	2.5 (-5.6, 10.6)	0.99
Gastrointestinal	1.5 (-6.1, 9.0)	0.99	-4.1 (-12.0, 3.8)	0.99	0.7 (-6.4, 7.8)	0.99	0.7 (-7.9, 9.3)	0.99

Genitourinary	1.5 (-5.2, 8.1)	0.99	-0.8 (-7.9, 6.2)	0.99	-0.4 (-6.7, 5.9)	0.99	0.3 (-7.2, 7.8)	0.99
Respiratory	1.7 (-5.0, 8.5)	0.99	-2.2 (-9.3, 4.9)	0.99	0.5 (-5.9, 6.8)	0.99	0.9 (-6.8, 8.5)	0.99

CI = confidence interval; ms = milliseconds; p-values reference the comparisons in HR, RR, rMSSD, SDNN and pNN50 between each cancer location relative to the non-cancer cases.

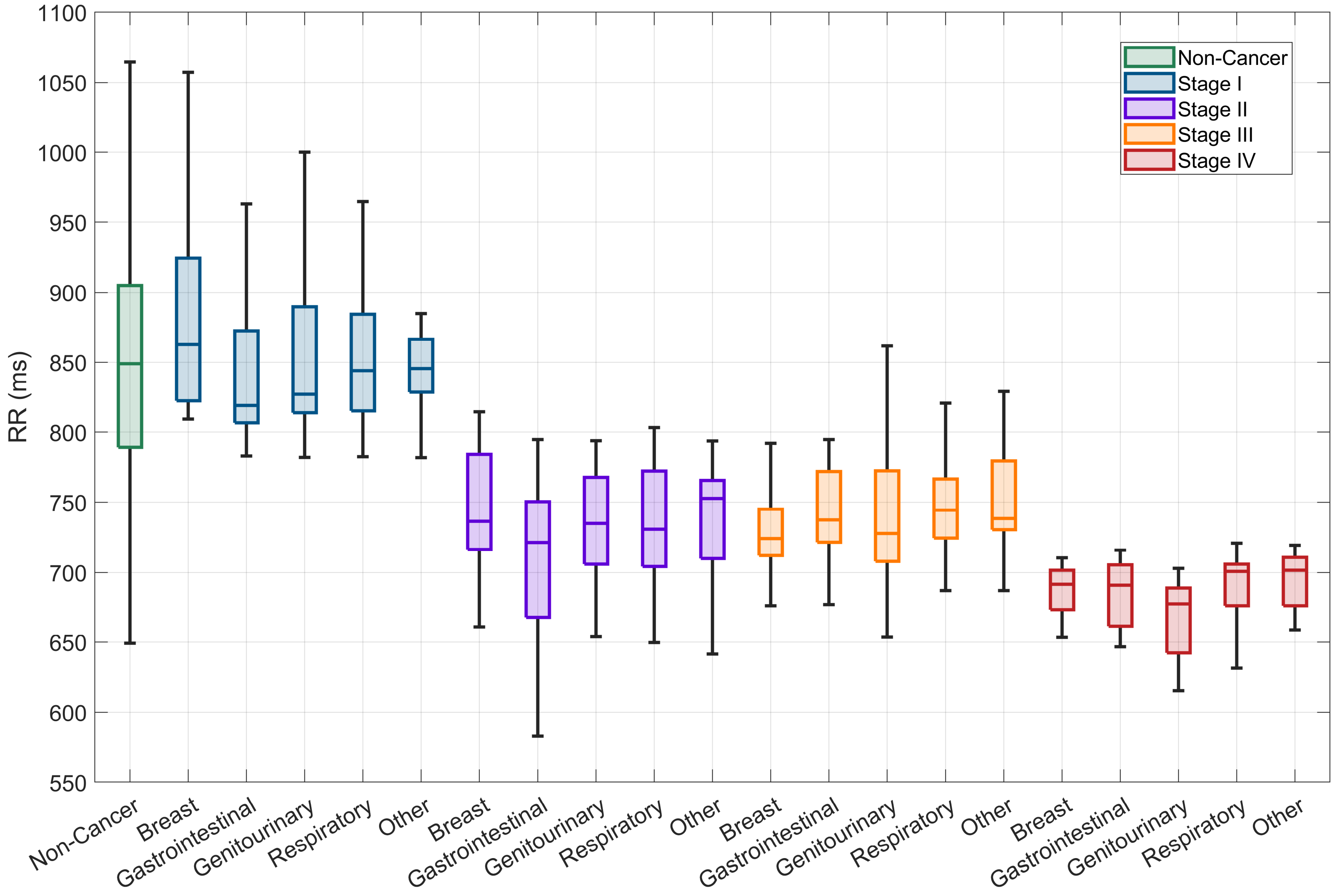

Figure S1. RR Intervals between Cancer and Non-Cancer Cases by Cancer Location and Stage.
